# Incremental Progress in the Enduring Conundrum to Provide an Off-the-Shelf Conduit for CABG That Can Compete With Native Grafts

**DOI:** 10.1016/j.jacbts.2025.101396

**Published:** 2025-10-27

**Authors:** AlleaBelle Bradshaw, Jennifer S. Lawton

**Affiliations:** Division of Cardiac Surgery, Department of Surgery, Johns Hopkins University, Baltimore, Maryland, USA

**Keywords:** CABG, regenerative medicine, spatial genomics, tissue engineering, vascular biology

**Figure:**
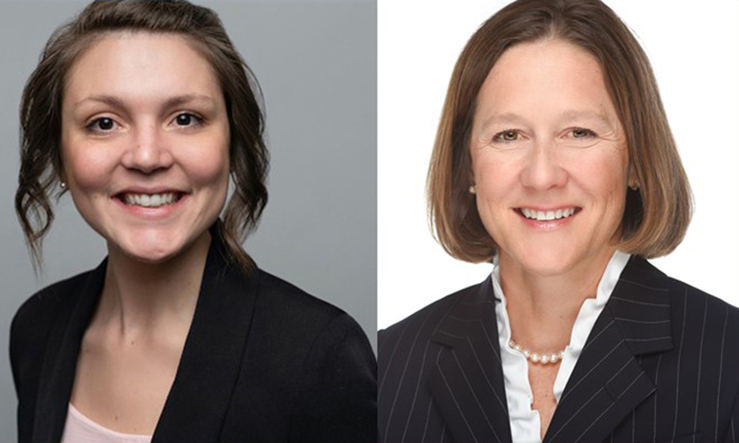

Approximately 160,000 patients undergo coronary artery bypass grafting (CABG) in the United States each year.^1^ CABG has become increasingly safe since it was developed in the 1960s, but graft failure remains a significant problem. Current guidelines recommend using the left internal mammary artery for stenosis of the left anterior descending artery and radial artery for the second most important target vessel.^2^ Despite these recommendations and data supporting total arterial grafting, the saphenous vein remains the most used conduit in the United States, which has a patency rate of 50% to 60% at 10 years.^3^ The possibility of using engineered vessels that do not depend on patient factors (eg, previous catheterization that precludes radial artery use) and have comparable patency rates could transform coronary artery revascularization.

In this issue of *JACC: Basic to Translational Science*, Williams et al^4^ use small-diameter acellular tissue engineered vessels (sdATEVs) comprised of human extracellular matrix proteins for CABG in 5 nonimmunosuppressed baboons with right coronary artery (RCA) ligation. The grafts in this study were sewn to the ascending aorta proximally, and distal to the ligated RCA. The grafts (3.5 mm) were significantly larger in diameter than the native target vessels (1.65 mm). Animals were given dual-antiplatelet therapy postoperatively and survived for 6 months. Several outcomes were studied, including clinically relevant variables (pulsatility indexes, postoperative cardiac function, and graft patency), as well as variables that provide insight into tissue biology (transcriptomics, histopathology, and wall shear stress). All 5 animals studied had patent grafts and good cardiac function. Interestingly, the grafts were recellularized with smooth muscle cells, a neomedia layer, and endothelial cells. Additionally, host cells derived from the native RCA media lined the graft lumen and expressed vascular smooth muscle cell and endothelial cell markers at 6 months. The sdATEV grafts were found to be narrowed after 6 months, approaching the diameter of the native vessel. The authors describe this as an adaptive remodeling, which may be true, although this is unconfirmed within this 6-month period. This finding could also represent detrimental neointimal hyperplasia, which is the known Achilles heel of saphenous vein grafts and is responsible for poor vein graft patency over time. Producing a conduit that can compete with the long-term patency of arterial grafts will be much more of a challenge.

By demonstrating feasibility in a clinically relevant large animal model, the authors pave the way for future studies that will investigate the efficacy of sdATEVs compared with current conduits. The goal will be for these nonautologous conduits to have comparable patency to autologous conduit. Future studies will include longer follow-up and models with atherosclerotic disease burden. Similar to bioprosthetic heart valves, demonstrating short-term follow-up is necessary but not sufficient, because most conduits will be patent after only 6 months. Saphenous vein grafts fail early for technical reasons and later because of neointimal hyperplasia (1 month to 1 year) followed by atherosclerosis (more than 1 year).^3^ Thus, longer follow-up is needed in translational models.

The authors only used grafts that were sewn to a native RCA that was completely ligated. Flow through the graft was thus optimized and not affected by competitive flow. The presence of coronary atherosclerotic disease, not present in this model using healthy animals, will also likely alter long-term patency results. Additional limitations include the inability to make conclusions based on only 5 animals with inconsistencies in cellular ingrowth and lymphocyte proliferation between animals, and a model that requires 8 weeks of cellularization of the vessel would not be immediately available at the time of surgery (and thus, not clinically relevant).

New surgical techniques in cardiac surgery have been supported by the demonstration of feasibility in translational large animal models before clinical trials in humans.^5^ Such models should mimic human anatomy and hemodynamics. Thus, the use of xenogeneic tissue adds an additional challenge, which is the need for a model with an immune response similar to that of humans. As the authors acknowledge, a major limitation for progress with acellular tissue engineered vessels (ATEVs) has been the absence of a fully translatable model. Decellularized human internal mammary artery grafts have been implanted into rabbits with 3-year follow-up and no signs of rejection; however, although rabbits tolerate the foreign tissue, they lack the anatomy and physiology needed for generalizability to human CABG (grafts were implanted into the rabbit femoral artery).^6^ The authors utilize an anatomically relevant model in the baboon^4^; however, the baboon mounts a more immunogenic response to the ATEV, precluding multiyear follow-up without immunosuppression. The ideal model appears to remain elusive.

The findings from this study are encouraging and support further investigation. There will certainly continue to be a need for immediately available (off-the-shelf) conduits in the foreseeable future in patients undergoing vascular and cardiac surgery. The potential human benefit of safe nonautologous grafts for CABG is worth the investment. Williams et al^4^ should be commended for their comprehensive evaluation of ATEVs within the limitations of a current primate model.
